# Supplementary material for: Scoping review of hearing loss attributed to congenital syphilis
Source: PLoS One. 2024 Apr 26;19(4):e0302452. doi: 10.1371/journal.pone.0302452 (PMC11051613; doi:10.1371/journal.pone.0302452)
Supplement: S1 Appendix — (DOCX) [file pone.0302452.s002.docx]

**Appendix 1**

**Ovid MEDLINE(R) ALL <1946 to October 14, 2021>**

| **#** | **Search Statement** | **Results** |
| --- | --- | --- |
| 1 | exp Syphilis/ or (Treponema pallidum or otosyphili* or syphili* or lues or luetic).mp. | 41124 |
| 2 | (HUTCHINSON'S TEETH or Hutchinson's triad).mp. | 13 |
| 3 | 1 or 2 | 41125 |
| 4 | exp Hearing Tests/ or exp Hearing/ or exp Hearing Disorders/ | 125228 |
| 5 | exp Vestibulocochlear Nerve/ or ("cranial nerve 8" or "cranial nerve eight" or "cranial nerve eighth" or "cranial nerve viii*" or ((statoacoustic or cochleovestibular or "vestibulocochlear" or "auditory vestibular") adj3 nerve*)).mp. | 15179 |
| 6 | exp Ear/ | 105521 |
| 7 | (hear or hearing or auditory or ears or ear or deaf or deafness or sound or sounds or hypoacusis).mp. | 440477 |
| 8 | exp Sound/ | 39935 |
| 9 | [audiology.mp](http://audiology.mp/). or exp Audiology/ | 4442 |
| 10 | 4 or 5 or 6 or 7 or 8 or 9 | 481307 |
| 11 | 3 and 10 | 626 |
| 12 | otosyphili*.mp. | 51 |
| 13 | 11 or 12 | 632 |
| 14 | exp Adult/ or (adult* or "senior citizen*" or man or man? or men or woman or women or elder* or adolescent* or adolescenc* or teen* or "high school*").mp. or ((old* or mature*) adj3 (patient* or person* or people*)).mp. | 10730482 |
| 15 | child/ or child, preschool/ or infant/ or infant, newborn/ or (child* or congenital or perinatal* or newborn* or infant or infants or baby or babies or fetus or foetus or fetal or foetal or "in utero" or "preterm" or "pre matur*" or prematur* or "young child*" or kindergarten* or elementary school* or toddler* or preschool* or "1 year old" or "2 year old" or "3 year old" or "4 year old" or "5 year old" or "6 year old" or "7 year old" or "8 year old" or "9 year old" or "10 year old" or "11 year old" or "12 year old").mp. | 3831187 |
| 16 | 14 and 15 | 1954237 |
| 17 | 14 not 16 | 8776245 |
| 18 | 13 not 17 | 375 |
| 19 | (Animal or animals or macaque* or rat or rats or mice or mouse or murine or rodent* or monkey or rabbit*).ti. or exp Animals/ | 24780943 |
| 20 | limit 19 to humans | 19769083 |
| 21 | 19 not 20 | 5011860 |
| 22 | 18 not 21 | 365 |

**Embase <1974 to 2021 October 14>**

| **#** | **Search Statement** | **Results** |
| --- | --- | --- |
| 1 | syphilis/ or (syphili* or lues or luetic or "treponema pallidum" or otosyphili*).mp. | 37658 |
| 2 | (HUTCHINSON'S TEETH or hutchinson's triad).mp. | 13 |
| 3 | 1 or 2 | 37660 |
| 4 | exp auditory system function/ or exp hearing test/ or [hearing.mp](http://hearing.mp/). | 260426 |
| 5 | exp vestibulocochlear nerve/ or ("cranial nerve 8" or "cranial nerve eight" or "cranial nerve eighth" or "cranial nerve viii*" or ((statoacoustic or cochleovestibular or "vestibulocochlear" or "auditory vestibular") adj3 nerve*)).mp. | 4203 |
| 6 | exp ear/ | 105336 |
| 7 | (hear or hearing or auditory or ears or ear or deaf or deafness or sound or sounds or hypoacusis).mp. | 539272 |
| 8 | [auditory.mp](http://auditory.mp/). | 202791 |
| 9 | exp sound/ | 219334 |
| 10 | [audiology.mp](http://audiology.mp/). or exp audiology/ [mp=title, abstract, heading word, drug trade name, original title, device manufacturer, drug manufacturer, device trade name, keyword heading word, floating subheading word, candidate term word] | 8958 |
| 11 | 4 or 5 or 6 or 7 or 8 or 9 or 10 | 764116 |
| 12 | 3 and 11 | 1120 |
| 13 | 3 and 12 | 1120 |
| 14 | exp adult/ or (adult* or "senior citizen*" or man or man? or men or woman or women or elder* or adolescent* or adolescenc* or teen* or "high school*").mp. or ((old* or mature*) adj3 (patient* or person* or people*)).mp. | 12973544 |
| 15 | (child* or congenital or perinatal* or newborn* or infant or infants or baby or babies or fetus or foetus or fetal or foetal or "in utero" or "preterm" or "pre matur*" or prematur* or "young child*" or kindergarten* or elementary school* or toddler* or preschool* or "1 year old" or "2 year old" or "3 year old" or "4 year old" or "5 year old" or "6 year old" or "7 year old" or "8 year old" or "9 year old" or "10 year old" or "11 year old" or "12 year old").mp. | 4372051 |
| 16 | 14 and 15 | 2179326 |
| 17 | 14 not 16 | 10794218 |
| 18 | 13 not 17 | 602 |
| 19 | exp animal/ or (animal or animals or macaque* or rat or rats or mice or mouse or murine or rodent* or monkey or rabbit*).ti. | 27841262 |
| 20 | human/ or [human.mp](http://human.mp/). | 23663582 |
| 21 | 19 and 20 | 23204487 |
| 22 | 19 not 21 | 4636775 |
| 23 | 18 not 22 | 597 |

**EBSCO CINAHL PLUS with Full Text**

Limiters/Expanders

Search modes - find all my search terms

| \| **#** \| **Query** \| **Results** \| \| --- \| --- \| --- \| \| S1 \| (MH "Syphilis+") \| 4,407 \| \| S2 \| ("Treponema pallidum" or otosyphili* or syphili* or lues or luetic) \| 6,489 \| \| S3 \| ("HUTCHINSON* TEETH" or "Hutchinson* triad") \| 3 \| \| S4 \| S1 OR S2 OR S3 \| 6,672 \| \| S5 \| (MH "Hearing+") \| 10,609 \| \| S6 \| (MH "Hearing Disorders+") \| 40,842 \| \| S7 \| (MH "Diagnosis, Ear+") \| 17,061 \| \| S8 \| (MH "Vestibulocochlear Nerve Diseases+") \| 2,412 \| \| S9 \| (MH "Vestibulocochlear Nerve+") \| 1,152 \| \| S10 \| ("cranial nerve 8" or "cranial nerve eight" or "cranial nerve eighth" or "cranial nerve viii*" or ((statoacoustic or cochleovestibular or "vestibulocochlear" or "auditory vestibular") N3 nerve*)) \| 560 \| \| S11 \| (MH "Ear+") \| 15,254 \| \| S12 \| (MH "Audiology") \| 6,072 \| \| S13 \| (hear or hearing or auditory or ears or ear or deaf or deafness or audiology or sounds or hypoacusis) \| 129,715 \| \| S14 \| (S5 OR S6 OR S7 OR S8 OR S9 OR S10 OR S11 OR S12 OR S13) \| 136,707 \| \| S15 \| S4 AND S14 \| 106 \| \| S16 \| (MH "Adult+") \| 1,933,874 \| \| S17 \| (MH "Adolescence+") \| 562,427 \| \| S18 \| (adult* or "senior citizen*" or man or men or woman or women or elder* or adolescent* or adolescenc* or teen* or "high school*") or ((old* or mature*) N3 (patient* or person* or people*)) \| 2,189,247 \| \| S19 \| S16 OR S17 OR S18 \| 2,609,106 \| \| S20 \| (MH "Child+") \| 711,581 \| \| S21 \| (child* or congenital or perinatal* or newborn* or infant or infants or baby or babies or fetus or foetus or fetal or foetal or "in utero" or "preterm" or "pre matur*" or prematur* or "young child*" or kindergarten* or elementary school* or toddler* or preschool* or "1 year old" or "2 year old" or "3 year old" or "4 year old" or "5 year old" or "6 year old" or "7 year old" or "8 year old" or "9 year old" or "10 year old" or "11 year old" or "12 year old") \| 1,085,566 \| \| S22 \| S20 OR S21 \| 1,085,566 \| \| S23 \| S19 AND S22 \| 507,551 \| \| S24 \| s19 NOT S23 \| 2,101,555 \| \| S25 \| s15 NOT s24 \| 54 \| |
| --- | --- | --- | --- | --- | --- | --- | --- | --- | --- | --- | --- | --- | --- | --- | --- | --- | --- | --- | --- | --- | --- | --- | --- | --- | --- | --- | --- | --- | --- | --- | --- | --- | --- | --- | --- | --- | --- | --- | --- | --- | --- | --- | --- | --- | --- | --- | --- | --- | --- | --- | --- | --- | --- | --- | --- | --- | --- | --- | --- | --- | --- | --- | --- | --- | --- | --- | --- | --- | --- | --- | --- | --- | --- | --- | --- | --- | --- | --- |

**SCOPUS Searched October 14, 2021 Results = 703**

(((TITLE-ABS-KEY(("Treponema pallidum" or otosyphili* or syphili* or lues or luetic or "HUTCHINSON'S TEETH" or "Hutchinson's triad"))) and ((TITLE-ABS-KEY(("cranial nerve 8" or "cranial nerve eight" or "cranial nerve eighth" or "cranial nerve viii*" or ((statoacoustic or cochleovestibular or "vestibulocochlear" or "auditory vestibular") w/3 nerve*)))) or (TITLE-ABS-KEY(hear OR hearing OR auditory OR ears OR ear OR deaf OR deafness OR sound OR sounds OR hypoacusis or audiology)))) AND NOT (((((mature or senior or elder* or old) w/3 (patient* or person or persons or people))) or (Adult* or man or man or men or women* or woman* or adolescen* or "high school*" or teen*)) and not (((((mature or senior or elder* or old) w/3 (patient* or person or persons or people))) or (Adult* or man or man or men or women* or woman* or adolescen* or "high school*" or teen*)) and ((child* or congenital or perinatal* or newborn* or infant or infants or baby or babies or fetus or foetus or fetal or foetal or "in utero" or "preterm" or "pre matur*" or prematur* or "pre school*" or "young child*" or kindergarten* or "elementary school*" or toddler* or preschool* or "1 year old" or "2 year old" or "3 year old" or "4 year old" or "5 year old" or "6 year old" or "7 year old" or "8 year old" or "9 year old" or "10 year old" or "11 year old" or "12 year old"))))) and Not (TITLE-ABS-KEY(animal or animals or rabbit* or rat or rats or mice or mouse or monkey or murine or macaque ) and not TITLE-ABS-KEY(human or humans))

**PROQUEST Dissertations and Theses Global Results =15**

((noft(("cranial nerve 8" OR "cranial nerve eight" OR "cranial nerve eighth" OR "cranial nerve viii*" OR hear OR hearing OR auditory OR ears OR ear OR deaf OR deafness OR sound OR sounds OR hypoacusis OR audiology)) OR noft(((statoacoustic OR cochleovestibular OR "vestibulocochlear" OR "auditory vestibular") w/3 nerve*))) AND noft("Treponema pallidum" OR otosyphili* OR syphili* OR lues OR luetic OR "HUTCHINSON'S TEETH" OR "Hutchinson's triad")) NOT (noft(rabbit*) OR noft(monkey) OR noft(cat) OR noft(cats) OR noft(dog) OR noft(dogs) OR noft(rat) OR noft(rats) OR noft(mice) OR noft(mouse) OR noft(murine))

**Cochrane Library Searched October 17, 2021 Results =11**

ID Search Hits

#1 MeSH descriptor: [Syphilis] explode all trees 155

#2 ("Treponema pallidum" or otosyphili* or syphili* or
 lues or luetic):ti,ab,kw 807

#3 (Hutchinson* next teeth or

Hutchinson* next triad):ti,ab,kw 0

#4 #1 or #2 or #3 808

#5 MeSH descriptor: [Hearing] explode all trees 431

#6 MeSH descriptor: [Hearing Tests] explode all trees 1044

#7 MeSH descriptor: [Hearing Disorders] explode all trees 1972

#8 MeSH descriptor: [Acupuncture, Ear] explode all trees 206

#9 MeSH descriptor: [Sound] explode all trees 701

#10 MeSH descriptor: [Audiology] explode all trees 16

#11 MeSH descriptor: [Vestibulocochlear Nerve]
explode all trees 39

#12 ("cranial nerve 8" or "cranial nerve eight" or "cranial nerve

eighth" or "cranial nerve viii"):ti,ab,kw 0

#13 (statoacoustic nerve* or cochleovestibular nerve*

or vestibulocochlear nerve or auditory vestibular nerve*):ti,ab,kw 19

#14 (hear or hearing or auditory or ears or ear or deaf or

deafness or sounds or hypoacusis or audiology):ti,ab,kw 21748

#15 #5 or #6 or #7 or #8 or #9 or #10 or #11 or
#12 or #13 or #14 22546

#16 #4 and #15 11

**PROSPERO Searched October 17, 2021 Results =9**

Line Search for Hits

#1 "Treponema pallidum" or otosyphili* or syphili* or lues or luetic or
"HUTCHINSON'S TEETH" or "Hutchinson's triad" 182

#2 "cranial nerve 8" or "cranial nerve eight" or
 "cranial nerve eighth" or "cranial nerve viii*" 1

#3 "statoacoustic nerve*" or "cochleovestibular nerve*" or
 "vestibulocochlear nerve " or "auditory vestibular nerve*" 9

#4 hear or hearing or auditory or ears or ear or deaf or deafness or
sounds or hypoacusis or audiology 4066

#5 #2 OR #3 OR #4 4067

#6 #1 AND #5 9
