# Supplementary material for: Scoping review of hearing loss attributed to congenital syphilis
Source: PLoS One. 2024 Apr 26;19(4):e0302452. doi: 10.1371/journal.pone.0302452 (PMC11051613; doi:10.1371/journal.pone.0302452)
Supplement: S2 Appendix — (DOCX) [file pone.0302452.s003.docx]

aPPENDIX 2

JBI Critical Appraisal Checklist for cohort studies

Reviewer ______________________________________ Date_______________________________

Author_______________________________________ Year_________ Record Number_________

|  | Yes | No | Unclear | Not applicable |
| --- | --- | --- | --- | --- |
| 1. Were the two groups similar and recruited from the same population? | □ | □ | □ | □ |
| 1. Were the exposures measured similarly to assign people to both exposed and unexposed groups? | □ | □ | □ | □ |
| 1. Was the exposure measured in a valid and reliable way? | □ | □ | □ | □ |
| 1. Were confounding factors identified? | □ | □ | □ | □ |
| 1. Were strategies to deal with confounding factors stated? | □ | □ | □ | □ |
| 1. Were the groups/participants free of the outcome at the start of the study (or at the moment of exposure)? | □ | □ | □ | □ |
| 1. Were the outcomes measured in a valid and reliable way? | □ | □ | □ | □ |
| 1. Was the follow up time reported and sufficient to be long enough for outcomes to occur? | □ | □ | □ | □ |
| 1. Was follow up complete, and if not, were the reasons to loss to follow up described and explored? | □ | □ | □ | □ |
| 1. Were strategies to address incomplete follow up utilized? | □ | □ | □ | □ |
| 1. Was appropriate statistical analysis used? | □ | □ | □ | □ |

Overall appraisal: Include □ Exclude □ Seek further info □

Comments (Including reason for exclusion)

________________________________________________________________________________________________________________________________________________________________________________________________

© JBI, 2020. All rights reserved. JBI grants use of these Critical Appraisal Checklist for Cohort Studies - **2**

tools for research purposes only. All other enquiries
should be sent to [jbisynthesis@adelaide.edu.au](mailto:jbisynthesis@adelaide.edu.au).

**JBI Critical Appraisal Checklist for Systematic Reviews and Research Syntheses**

Reviewer Date

| Author Year Record Number | Yes No | | | Unclear | | Not applicable | |
| --- | --- | --- | --- | --- | --- | --- | --- |
| 1. Is the review question clearly and explicitly stated? | | □ | □ | | □ | | □ |
| 2. Were the inclusion criteria appropriate for the review question? | | □ | □ | | □ | | □ |
| 3. Was the search strategy appropriate? | | □ | □ | | □ | | □ |
| 4. Were the sources and resources used to search for studies adequate? | | □ | □ | | □ | | □ |
| 5. Were the criteria for appraising studies appropriate? | | □ | □ | | □ | | □ |
| 6. Was critical appraisal conducted by two or more reviewers independently? | | □ | □ | | □ | | □ |
| 7. Were there methods to minimize errors in data extraction? | | □ | □ | | □ | | □ |
| 8. Were the methods used to combine studies appropriate? | | □ | □ | | □ | | □ |
| 9. Was the likelihood of publication bias assessed? | | □ | □ | | □ | | □ |
| 10. Were recommendations for policy and/or practice supported by the reported data? | | □ | □ | | □ | | □ |
| 11. Were the specific directives for new research appropriate? | | □ | □ | | □ | | □ |

Overall appraisal: Include □ Exclude □ Seek further info □

Comments (Including reason for exclusion

**JBI Critical Appraisal Checklist for Case Reports**

Reviewer Date

| Author Year Record Number | Yes | No | Unclear NA | |
| --- | --- | --- | --- | --- |
| 1. Were patient’s demographic characteristics clearly described? | □ | □ | □ | □ |
| 2. Was the patient’s history clearly described and presented as a timeline? | □ | □ | □ | □ |
| 3. Was the current clinical condition of the patient on presentation clearly described? | □ | □ | □ | □ |
| 4. Were diagnostic tests or assessment methods and the results clearly described? | □ | □ | □ | □ |
| 5. Was the intervention(s) or treatment procedure(s) clearly described? | □ | □ | □ | □ |
| 6. Was the post-intervention clinical condition clearly described? | □ | □ | □ | □ |
| 7. Were adverse events (harms) or unanticipated events identified and described? | □ | □ | □ | □ |
| 8. Does the case report provide takeaway lessons? | □ | □ | □ | □ |

Overall appraisal: Include □ Exclude □ Seek further info □

Comments (Including reason for exclusion

**Joanna Briggs Institute 2017 Critical Appraisal Checklist for Case Reports**

**JBI critical appraisal checklist for studies reporting prevalence data**

Reviewer Date

| Author Year Record Number Yes | | No | Unclear | Not applicable | |
| --- | --- | --- | --- | --- | --- |
| 1. Was the sample frame appropriate to address the target population? | □ | □ | □ | | □ |
| 2. Were study participants sampled in an appropriate way? | □ | □ | □ | | □ |
| 3. Was the sample size adequate? | □ | □ | □ | | □ |
| 4. Were the study subjects and the setting described in detail? | □ | □ | □ | | □ |
| 5. Was the data analysis conducted with sufficient coverage of the identified sample? | □ | □ | □ | | □ |
| 6. Were valid methods used for the identification of the condition? | □ | □ | □ | | □ |
| 7. Was the condition measured in a standard, reliable way for all participants? | □ | □ | □ | | □ |
| 8. Was there appropriate statistical analysis? | □ | □ | □ | | □ |
| 9. Was the response rate adequate, and if not, was the low response rate managed appropriately? | □ | □ | □ | | □ |

Overall appraisal: Include □ Exclude □ Seek further info □

Comments (Including reason for exclusion)

**Joanna Briggs Institute 2017 Critical Appraisal Checklist for Prevalence Studies**

**JBI Critical Appraisal Checklist for Case Series**

Reviewer ______________________________________ Date_______________________________

Author_______________________________________ Year_________ Record Number_________

|  | Yes | No | Unclear | Not applicable |
| --- | --- | --- | --- | --- |
| - Were there clear criteria for inclusion in the case series? | □ | □ | □ | □ |
| - Was the condition measured in a standard, reliable way for all participants included in the case series? | □ | □ | □ | □ |
| - Were valid methods used for identification of the condition for all participants included in the case series? | □ | □ | □ | □ |
| - Did the case series have consecutive inclusion of participants? | □ | □ | □ | □ |
| - Did the case series have complete inclusion of participants? | □ | □ | □ | □ |
| - Was there clear reporting of the demographics of the participants in the study? | □ | □ | □ | □ |
| - Was there clear reporting of clinical information of the participants? | □ | □ | □ | □ |
| - Were the outcomes or follow up results of cases clearly reported? | □ | □ | □ | □ |
| - Was there clear reporting of the presenting site(s)/clinic(s) demographic information? | □ | □ | □ | □ |
| - Was statistical analysis appropriate? | □ | □ | □ | □ |

Overall appraisal: Include □ Exclude □ Seek further info □

Comments (Including reason for exclusion)

________________________________________________________________________________________________________________________________________________________________________________________________

© JBI, 2020. All rights reserved. JBI grants use of these Critical Appraisal Checklist for Case Series - **3**

tools for research purposes only. All other enquiries
should be sent to [jbisynthesis@adelaide.edu.au](mailto:jbisynthesis@adelaide.edu.au).
