# Supplementary material for: Scoping review of hearing loss attributed to congenital syphilis
Source: PLoS One. 2024 Apr 26;19(4):e0302452. doi: 10.1371/journal.pone.0302452 (PMC11051613; doi:10.1371/journal.pone.0302452)
Supplement: Attachment — Submitted filename: PONE-D-23-32597_reviewer.pdf [file pone.0302452.s004.pdf]

Title of the Manuscript:
"Scoping Review of hearing loss attributed to congenital syphilis."

Overall Evaluation:
The paper offers valuable insights into hearing loss attributed to congenital syphilis. The authors put a lot of effort into finding the relevant literature and conducting the scoping review. Overall, I find the work is satisfactory. However, I find changes are required to the text. There were a few spelling and grammar mistakes. I recommend improving the organization of the content in the paper.

Major Contributions:
Relevance of Topic: The topic is of high importance and is aptly chosen.

Comprehensiveness: The review covers a wide array of studies and provides a comprehensive overview.

Originality: The topic that the authors selected is a novel research area. There is adequate information however I suggest improvements to further identify gaps and mention potential areas for future research.

Areas for Improvement:
I used the bellow table and added my suggestions for each section for improvement can be found through the attached table.

Summary and Recommendation:
Overall, this manuscript is a commendable effort in synthesizing the current knowledge on hearing loss attributed to congenital syphilis. It provides an insightful overview, though some areas require refinement for clarity and depth. With revisions, particularly in the introduction, methodology, and discussion sections, this manuscript will be a valuable addition to the literature. I recommend acceptance of the manuscript after revisions.

Preferred Reporting Items for Systematic reviews and Meta-Analyses extension for Scoping Reviews (PRISMA-ScR) Checklist

SECTION	ITEM	PRISMA-ScR CHECKLIST ITEM	REVIEW
**TITLE**			
Title	1	Identify the report as a scoping review.	Correctly identified
**ABSTRACT**			
Structured summary	2	Provide a structured summary that includes (as applicable): background, objectives, eligibility criteria, sources of evidence, charting methods, results, and conclusions that relate to the review questions and objectives.	The abstract is adequate
**INTRODUCTION**			
Rationale	3	Describe the rationale for the review in the context of what is already known. Explain why the review	The rationale was clear

SECTION	ITEM	PRISMA-ScR CHECKLIST ITEM	REVIEW
		questions/objectives lend themselves to a scoping review approach.	
Objectives	4	Provide an explicit statement of the questions and objectives being addressed with reference to their key elements (e.g., population or participants, concepts, and context) or other relevant key elements used to conceptualize the review questions and/or objectives.	An explicit statement of research questions and objectives is not provided. I recommend rethinking and clearly rewriting the text. Please use bullet points and proper formatting. It helps the reader to understand clearly.
**METHODS**			
Protocol and registration	5	Indicate whether a review protocol exists; state if and where it can be accessed (e.g., a Web address); and if available, provide registration information, including the registration number.	No
Eligibility criteria	6	Specify characteristics of the sources of evidence used as eligibility criteria (e.g., years considered, language, and publication status), and provide a rationale.	A rationale is provided
Information sources*	7	Describe all information sources in the search (e.g., databases with dates of coverage and contact with authors to identify additional sources), as well as the date the most recent search was executed.	Information sources are included
Search	8	Present the full electronic search strategy for at least 1 database, including any limits used, such that it could be repeated.	Details were found from the appendix
Selection of sources of evidence†	9	State the process for selecting sources of evidence (i.e., screening and eligibility) included in the scoping review.	The selection process is clear. However, I recommend re-think and clearly describe in the text. Use bullet points and proper formatting. Formatting issues were identified.
Data charting process‡	10	Describe the methods of charting data from the included sources of evidence (e.g., calibrated forms or forms that have been tested by the team before their use, and whether data charting was done independently or in duplicate) and any processes for obtaining and confirming data from investigators.	Data charting process were found.
Data items	11	List and define all variables for which data were sought and any assumptions and simplifications made.	Described in the text
Critical appraisal of individual sources of evidence§	12	If done, provide a rationale for conducting a critical appraisal of included sources of evidence; describe the methods used and how this	Authors report they used JBI critical appraisal tool. Information was found through Appendix 2.

SECTION	ITEM	PRISMA-ScR CHECKLIST ITEM	REVIEW
		information was used in any data synthesis (if appropriate).	
Synthesis of results	13	Describe the methods of handling and summarizing the data that were charted.	Described in the text
**RESULTS**			
Selection of sources of evidence	14	Give numbers of sources of evidence screened, assessed for eligibility, and included in the review, with reasons for exclusions at each stage, ideally using a flow diagram.	The flow diagram is included. It is clear.
Characteristics of sources of evidence	15	For each source of evidence, present characteristics for which data were charted and provide the citations.	Charts are included. I recommend moving the charts below to relevant sections in the text. Otherwise, it was challenging to scroll down and go back to the text to relate what the authors are trying to describe.
Critical appraisal within sources of evidence	16	If done, present data on critical appraisal of included sources of evidence (see item 12).	Provided
Results of individual sources of evidence	17	For each included source of evidence, present the relevant data that were charted that relate to the review questions and objectives.	provided
Synthesis of results	18	Summarize and/or present the charting results as they relate to the review questions and objectives.	A short description. As I mentioned earlier the research questions are unclear and it would make sense if each results section is described relevant to the research questions that he authors wants to answer.
**DISCUSSION**			
Summary of evidence	19	Summarize the main results (including an overview of concepts, themes, and types of evidence available), link to the review questions and objectives, and consider the relevance to key groups.	Found complex sentence structures. Look into the text again and think of clear explanation.
Limitations	20	Discuss the limitations of the scoping review process.	I recommend discussing some limitations based on the following topics. If you have already discussed them, please ignore.<br>1. Breadth Over Depth<br>2. Quality Assessment<br>3. Heterogeneity of Data<br>4. Time and Resource Intensive<br>5. Publication Bias<br>6. Language Bias<br>7. Scope Creep<br>8. Update Challenges

SECTION	ITEM	PRISMA-ScR CHECKLIST ITEM	REVIEW
			9. Lack of Methodological Standardization<br>10. Interpretation of Results
Conclusions	21	Provide a general interpretation of the results with respect to the review questions and objectives, as well as potential implications and/or next steps.	Adequate
**FUNDING**			
Funding	22	Describe sources of funding for the included sources of evidence, as well as sources of funding for the scoping review. Describe the role of the funders of the scoping review.	Disclosed

PLOS ONE

Scoping review of hearing loss attributed to congenital syphilis.

--Manuscript Draft--

**Manuscript Number:**	PONE-D-23-32597
**Article Type:**	Research Article
**Full Title:**	Scoping review of hearing loss attributed to congenital syphilis.
**Short Title:**	Scoping review of hearing loss attributed to congenital syphilis.
**Corresponding Author:**	Joan L Robinson<br>University of Alberta<br>Edmonton, AB CANADA
**Keywords:**	congenital syphilis; hearing loss
**Abstract:**	Objectives: There are no narrative or systematic reviews of hearing loss in patients with congenital syphilis. The aim of this study was to perform a scoping review to determine what is known about the incidence, characteristics, prognosis, and therapy of hearing loss in children or adults with presumed congenital syphilis.<br>Design: PROSPERO, OVID Medline, OVID EMBASE, Cochrane Library (CDSR and Central), Proquest Dissertations and Theses Global, and SCOPUS were searched from inception to March 31, 2023. Articles were included if patients with hearing loss were screened for CS, ii) patients with CS were screened for hearing loss, iii) they were case reports or case series that describe the characteristics of hearing loss, or iv) an intervention for hearing loss attributed to CS was studied.<br>Results: Thirty-six articles met the inclusion criteria. Five reported an incidence of CS in 0.3% to 8% of children with hearing loss, but all had a high risk of bias. Seven reported that 0 to 19% of children with CS had hearing loss, but the only one with a control group showed comparable rates in cases and controls. There were 18 case reports/ case series (one of which also reported screening children with hearing loss for CS), reporting that onset of hearing loss was usually during adolescence or adulthood. The 7 intervention studies were all uncontrolled and published in 1983 or earlier and reported variable results with penicillin, prednisone, and/or ACTH.<br>Conclusions: The current literature is not informative with regard to the incidence, characteristics, prognosis, and therapy of hearing loss in children or adults with presumed congenital syphilis.
**Order of Authors:**	Aleena Hafeez
	Karina Cavalcanti
	Zaharadeen Jimoh
	Francesca Seal
	Joan L Robinson
	Nahla Gomaa
**Additional Information:**	
**Question**	**Response**
**Financial Disclosure**<br><br>Enter a financial disclosure statement that describes the sources of funding for the work included in this submission. Review the submission guidelines for detailed requirements. View published research articles from *PLOS ONE* for specific examples.	The author(s) received no specific funding for this work.

This statement is required for submission and **will appear in the published article** if the submission is accepted. Please make sure it is accurate.<br><br>**Funded studies**<br>Enter a statement with the following details:<br>• Initials of the authors who received each award<br>• Grant numbers awarded to each author<br>• The full name of each funder<br>• URL of each funder website<br>• Did the sponsors or funders play any role in the study design, data collection and analysis, decision to publish, or preparation of the manuscript?<br><br>Did you receive funding for this work?	
**Competing Interests**<br><br>Use the instructions below to enter a competing interest statement for this submission. On behalf of all authors, disclose any competing interests that could be perceived to bias this work—acknowledging all financial support and any other relevant financial or non-financial competing interests.<br><br>This statement is required for submission and **will appear in the published article** if the submission is accepted. Please make sure it is accurate and that any funding sources listed in your Funding Information later in the submission form are also declared in your Financial Disclosure statement.<br><br>View published research articles from *PLOS ONE* for specific examples.	The authors have declared that no competing interests exist.

**NO authors have competing interests**<br><br>Enter: *The authors have declared that no competing interests exist.*<br><br>**Authors with competing interests**<br><br>Enter competing interest details beginning with this statement:<br><br>*I have read the journal's policy and the authors of this manuscript have the following competing interests: [insert competing interests here]*<br><br>* typeset	
**Ethics Statement**<br><br>Enter an ethics statement for this submission. This statement is required if the study involved:<br><br>• Human participants<br>• Human specimens or tissue<br>• Vertebrate animals or cephalopods<br>• Vertebrate embryos or tissues<br>• Field research<br><br>Write "N/A" if the submission does not require an ethics statement.<br><br>General guidance is provided below. Consult the submission guidelines for detailed instructions. **Make sure that all information entered here is included in the Methods section of the manuscript.**	N/A

**Format for specific study types**<br><br>**Human Subject Research (involving human participants and/or tissue)**<br>• Give the name of the institutional review board or ethics committee that approved the study<br>• Include the approval number and/or a statement indicating approval of this research<br>• Indicate the form of consent obtained (written/oral) or the reason that consent was not obtained (e.g. the data were analyzed anonymously)<br><br>**Animal Research (involving vertebrate animals, embryos or tissues)**<br>• Provide the name of the Institutional Animal Care and Use Committee (IACUC) or other relevant ethics board that reviewed the study protocol, and indicate whether they approved this research or granted a formal waiver of ethical approval<br>• Include an approval number if one was obtained<br>• If the study involved *non-human primates*, add *additional details* about animal welfare and steps taken to ameliorate suffering<br>• If anesthesia, euthanasia, or any kind of animal sacrifice is part of the study, include briefly which substances and/or methods were applied<br><br>**Field Research**<br><br>Include the following details if this study involves the collection of plant, animal, or other materials from a natural setting:<br>• Field permit number<br>• Name of the institution or relevant body that granted permission	
**Data Availability**<br><br>Authors are required to make all data underlying the findings described fully available, without restriction, and from the time of publication. PLOS allows rare exceptions to address legal and ethical concerns. See the PLOS Data Policy and FAQ for detailed information.	Yes - all data are fully available without restriction

Powered by Editorial Manager® and ProduXion Manager® from Aries Systems Corporation

A Data Availability Statement describing where the data can be found is required at submission. Your answers to this question constitute the Data Availability Statement and **will be published in the article**, if accepted.<br><br>**Important:** Stating 'data available on request from the author' is not sufficient. If your data are only available upon request, select 'No' for the first question and explain your exceptional situation in the text box.<br><br>Do the authors confirm that all data underlying the findings described in their manuscript are fully available without restriction?	
**Describe where the data may be found in full sentences. If you are copying our sample text, replace any instances of XXX with the appropriate details.**<br><br>• If the data are **held or will be held in a public repository**, include URLs, accession numbers or DOIs. If this information will only be available after acceptance, indicate this by ticking the box below. For example: *All XXX files are available from the XXX database (accession number(s) XXX, XXX.)*.<br>• If the data are all contained **within the manuscript and/or Supporting Information files**, enter the following: *All relevant data are within the manuscript and its Supporting Information files.*<br>• If neither of these applies but you are able to provide **details of access elsewhere**, with or without limitations, please do so. For example:<br><br>*Data cannot be shared publicly because of [XXX]. Data are available from the XXX Institutional Data Access / Ethics Committee (contact via XXX) for researchers who meet the criteria for access to confidential data.*<br><br>*The data underlying the results presented in the study are available from (include the name of the third party*	This is a systematic review so all data can be derived from the included manuscripts.

and contact information or URL).<br>• This text is appropriate if the data are owned by a third party and authors do not have permission to share the data.<br><br>* typeset	
Additional data availability information:	

Manuscript

Click here to access/download;Manuscript;Scoping review Oct 6.docx

Scoping Review of hearing loss attributed to congenital syphilis

Aleena A Hafeez[1], Karina C B Cavalcanti[2], Zaharadeen Jimoh[3], Francesca B. Seal[4], Joan L. Robinson[5,] Nahla A. Gomaa[6]

[1] Department of Pediatrics, Queen's University, Kingston, Ontario, Canada

[2] Research investigator, University of Alberta, Edmonton, Alberta, Canada

[3] Department of Nursing, University of Alberta, Edmonton, Alberta, Canada

[4] Department of Anesthesiology, University of Alberta, Edmonton, Alberta, Canada.

[5] Department of Pediatrics, University of Alberta, Edmonton, Alberta, Canada

[6] Division of Otolaryngology-Head & Neck Surgery, University of Alberta, Edmonton, Alberta, Canada

Financial disclosures/conflicts of interest:
None for any authors

All correspondence should be addressed to:
Joan L Robinson, 3-556 ECHA, 11405-87 Ave, Edmonton, AB, T6G 1C9
Email: jr3@ualberta.ca
Phone: (780) 248-5528

Abstract

Objectives: There are no narrative or systematic reviews of hearing loss in patients with congenital syphilis. The aim of this study was to perform a scoping review to determine what is known about the incidence, characteristics, prognosis, and therapy of hearing loss in children or adults with presumed congenital syphilis.

Design: PROSPERO, OVID Medline, OVID EMBASE, Cochrane Library (CDSR and Central), Proquest Dissertations and Theses Global, and SCOPUS were searched from inception to March

what is number (i)

31, 2023. Articles were included if patients with hearing loss were screened for CS, ii) patients with CS were screened for hearing loss, iii) they were case reports or case series that describe the characteristics of hearing loss, or iv) an intervention for hearing loss attributed to CS was studied.

Results: Thirty-six articles met the inclusion criteria. Five reported an incidence of CS in 0.3% to 8% of children with hearing loss, but all had a high risk of bias. Seven reported that 0 to 19% of children with CS had hearing loss, but the only one with a control group showed comparable rates in cases and controls. There were 18 case reports/ case series (one of which also reported screening children with hearing loss for CS), reporting that onset of hearing loss was usually during adolescence or adulthood. The 7 intervention studies were all uncontrolled and published in 1983 or earlier and reported variable results with penicillin, prednisone, and/or ACTH.

Conclusions: The current literature is not informative with regard to the incidence, characteristics, prognosis, and therapy of hearing loss in children or adults with presumed congenital syphilis.

Introduction

Syphilis is a sexually transmitted infection caused by the bacterium *Treponema pallidum*. (Ref) If not recognized and treated early in pregnancy, fetal transmission can occur (Public Health Agency of Canada, 2014). According to the international Joint Committee on Infant Hearing, congenital syphilis (CS) is a risk indicator for hearing loss (Joint Committee on Infant Hearing, 2007, 2019).

Double check if the statistics are correct

For decades, congenital syphilis had almost disappeared in Canada and the United States due to low rates of syphilis in the community and universal prenatal screening. In 2021 in Canada, the incidence of infectious syphilis in females was 729% higher than in 2017. The number of cases of confirmed early congenital syphilis rose from 7 in 2017 to 96 in 2021 (Public Health Agency of Canada, 2022). Trends in the US mirror this explosion in the incidence of CS, with an increase of 464% from 2001 to 2021 (Centers for Disease Control and Prevention, 2021).

Review questions are unclear

The recent resurgence has increased interest in the clinical manifestations and complications of CS. There are no published data on the incidence or characteristics of hearing loss from CS. We therefore performed a scoping review to better characterize this entity. This will inform the studies that need to be done to determine the incidence and age of onset of hearing loss from CS, the severity of hearing loss, and interventions that warrant further study.

better is a vague word

Methods

The methodology was based on the Preferred Reporting Items for a Systematic Review and Meta-analysis of Diagnostic Test Accuracy Studies: The PRISMA-DTA statement (Page et al., 2021). A search was executed by a health librarian on the following databases: PROSPERO, OVID Medline, OVID EMBASE, Cochrane Library (CDSR and Central), Proquest Dissertations

and Theses Global, and SCOPUS using controlled vocabulary (eg: MeSH, Emtree, etc) and

where are the keywords?

selecting key words representing the concepts “congenital syphilis" or "hearing loss” (Appendix 1). Databases were searched from inception to October 17, 2021 with an updated search to March 31, 2023.

Articles were included if they described persons of any age with hearing loss that the authors of the article attributed to CS. To delineate the burden and incidence of hearing loss from CS, we included any studies that i) screened children with hearing loss for evidence of CS or ii) screened children with CS for hearing loss. We also included randomized controlled trials (RCTs), cross-sectional studies, case series, and case reports that described the characteristics of hearing loss, the long-term outcomes of hearing loss, or the results of any interventions for hearing loss. We excluded autopsy reports, animal studies, studies focusing solely on acquired syphilis and those published in a language other than English, French, or Portuguese.

Might not need to included this information

Articles published in English were screened by two reviewers independently [AH, KC],

author or autors?

and conflicts were resolved by a senior author [JR, NG]. Articles published in French had a single reviewer [FS]. There were no Portuguese articles. Because of the small number of recent articles, preprints were included. The protocol has not been published.

PROSPERO?

Studies were divided into four types: i) those that screened patients with hearing loss for CS, ii) those that screened patients with CS for hearing loss, iii) case reports or case series that

related to CS?

describe the characteristics of hearing loss, and iv) studies that describe an intervention for

Research Electronic Data Capture

hearing loss. Data were collected and managed using REDCap electronic data capture tools (Harris et al., 2019) hosted at the University of Alberta with the extracted data determined by the study type.

The JBI critical appraisal tool was used as appropriate to assess all included studies (Joanna Briggs, 2017; Moola S, 2020; Munn Z, 2020; Whiting et al., 2016) (Appendix 2). The critical appraisal and bias risk assessment was completed by a single reviewer [NG], and all were rated as low, high, or unclear risk of bias.

Results

The search yielded 1983 records of which 832 were duplicates. Screening led to 154 records for full-text review of which 36 met inclusion criteria (Figure 1). Figure 1 is far away from the text

Screening of patients with hearing loss for congenital syphilis

Modify the sentence

There were 5 studies published from 1900 to 1990 (Table 1; Table 2), all with high risk of bias. The incidence of CS ranged from 0.3% to 8% in children attending schools for the hearing impaired and was 2% in children seen at a clinic for the hearing impaired.

Screening of patients with CS for hearing loss

There were 7 studies of which 4 were published from 2016 to 2022 (Table 3). Risk of bias was low for 3, unclear for 3, and high for 1. Hearing loss was reported in 0 to 19% of children with probable or proven CS . One study from the modern era showed an incidence of

Not required or explain in words

6% (22/342) (Besen et al., 2022). However, a small recent study reported no hearing loss for 7 infants treated in utero, a 5% incidence for 37 treated at birth, and a 6% incidence in 49 controls (Aguiar et al., 2022).

Case series and case reports

There were 10 case series (one of which was also included in Table 2) (Table 4A) and 8 case reports (Table 4B) of which all but 6 were published prior to 1980. The risk of bias was low for 10 articles, unclear for 3 and high for 5. Hearing loss was often first noted in adolescence or adulthood with the youngest being 5 years old at diagnosis (Khetarpal et al., 2011). Many

cases also had interstitial keratitis. A wide variety of therapies are reported with small numbers of patients and inconsistent results that were often subjective.

Interventions for hearing loss

The 7 studies included 6 to 39 patients with the most recent one being from 1983 (Table 5). All were observational and typically included a penicillin with addition of prednisone and then ACTH if response was poor or transient. Outcomes were often subjective and inconsistent. Risk of bias was low for 2 studies and unclear for 5.

Discussion

The scoping review shows that the literature on hearing loss due to CS is immature. All but one reported as a pre-print (Aguiar et al., 2022) are observational studies and only 15 of 36 (42%) were at low risk of bias.

complex sentence please break it down

As expected, there were major variations in the study methodologies employed to diagnose hearing loss when comparing the early 1900s, where investigators used basic tuning fork tests and subjective behavioral responses (Fraser, 1916), to studies performed after the year 2000, which used full diagnostic tests or Auditory Brainstem Responses (ABR) for neonates (Lim et al., 2021).

A small percentage of children attending schools for the hearing impaired had evidence of CS, but this information is of limited value without a control group from the same jurisdiction. The percentage of hearing loss that is due to CS no doubt varies considerably by country and over time.

It is perhaps unexpected that almost all case reports and case series describe recognition of hearing loss only in adolescence or adulthood. It is possible that hearing loss started years prior but was not recognized. The major problem with all these reports is that they do not

exclude the possibility that the patient had acquired syphilis or had another etiology for their hearing loss.

Clearly, there is paucity of up-to-date literature regarding this health problem. The majority of articles were published before 1980. The recent surge in CS cases in Canada and the United States may lead to further studies. Results from neonatal hearing screening programs in underprivileged countries where the incidence of CS never waned are informative. Besen Which year? screened 21,434 newborns in Brazil and reported a prevalence of test failure in the Universal Neonatal Hearing Screening Program (UNHS) of 1.6% (95% CI: 1.4; 1.8) using Otoacoustic Emission and ABR to identify both cochlear and retrocochlear damage. They report that 1.7% (95% CI: 1.5; 1.8) had congenital syphilis but do not report how many with CS had hearing loss (Besen et al., 2022). In a follow-up report of 34,801 infants screened 2017 through 2021, they report that neonates with CS were 2.38 times as likely to fail in the UNHS as those without CS (Besen et al., 2023). However, another small study from Brazil reported as a pre-print examined failed hearing screens at 2 months of life did not find an association between CS and failed hearing screens (Aguiar et al., 2022).

As for interventions, antibiotics to treat CS were presumably always given at the time of diagnosis of hearing loss if the patient had not previously been adequately treated. There are no convincing reports that this alone resulted in sustained improved hearing. Uncontrolled studies that included corticosteroids with or without ACTH reported variable response and improvement in hearing was often subjective.

The main limitation of the study is the lack of high quality studies.

Conclusion

Our study outlines a general map of the trend of publications across the decades and shows that the incidence of hearing loss due to CS is completely unknown. Until there are high quality long-term observational studies, it is difficult to know what hearing screening to recommend for children with CS. Onset is often in adolescence or adulthood. Therefore, there is a need to increase awareness that people of all ages with unexplained hearing loss of sudden or gradual onset should be screened for syphilis. Other than treatment of the CS, no other treatments can be recommended until there are RCTs, or cohort studies with valid control groups.

Funding

No funding was obtained for this study.

References

Aguiar, L. B., da Cunha, B. K., Bezerra, M.T., de Brito, M. E., Arrais N. M., Moore., D. R., Balen, S.A. (2022). Follow-up of neonatal screening using automatic auditory brainstem responses in infants with congenital syphilis: prospective cross-sectional study. https://doi.org/10.21203/rs.3.rs-1564783/v1

Baron, F., & Déjour, B. (1967). [Deafness and late congenital syphilis]. *Revue de laryngologie - otologie - rhinologie*, *88*(11), 877-883.

Besen, E., Paiva, K. M., Cigana, L. B., Machado, M. J., Samelli, A. G., & Haas, P. (2023). Prevalence of Congenital Infections in Newborns and Universal Neonatal Hearing Screening in Santa Catarina, Brazil. *Audiology Research*, *13*(1), 107-115.

Besen, E., Paiva, K. M., Hillesheim, D., Cigana, L. B., & Haas, P. (2022). Congenital syphilis associated with hearing screening failure in southern Brazilian newborns. *Brazilian Journal of Otorhinolaryngology*, *88*, S20-S24. https://doi.org/10.1016/j.bjorl.2021.07.003

Centers for Disease Control and Prevention, T. (2021). *Sexually Transmitted Disease Surveillance 2021*. US: Centers for Disease Control and Prevention Retrieved from https://www.cdc.gov/std/statistics/2021/default.htm#print

Dawkins, R. S., Sharp, M., & Morrison, A. W. (1968). Steroid treatment in congenital syphilitic deafness. *The Journal of laryngology and otology*, *82*(12), 1095-1107.

Dobbin, J. M., & Perkins, J. H. (1983). Otosyphilis and hearing loss: response to penicillin and steroid therapy. *The Laryngoscope*, *93*(12), 1540-1543.

Fraser, J. S. (1916). Congenital Syphilitic Disease of the Ear. *Proceedings of the Royal Society of Medicine*, *9*(Otol Sect), 62-74.

Ganga, N., Rajagopal, B., Rajendran, S., & Padmanabhan, A. S. (1991). Deafness in children--an analysis. *Indian pediatrics*, *28*(3), 273-276.

Gleich, L. L., Urbina, M., & Pincus, R. L. (1994). Asymptomatic congenital syphilis and auditory brainstem response. *International journal of pediatric otorhinolaryngology*, *30*(1), 11-13.

Gururaj Kumar, A., Lakshmi, N., & Sudhakar Babu, V. (1990). The incidence of congenital syphilis and hepatitis-B virus surface antigen carrier state among deaf mute children. *Indian Journal of Dermatology, Venereology and Leprology*, *56*(1), 25-26.

Harris, P. A., Taylor, R., Minor, B. L., Elliott, V., Fernandez, M., O'Neal, L., . . . Duda, S. N. (2019). The REDCap consortium: Building an international community of software platform partners. *J Biomed Inform*, *95*, 103208. https://doi.org/10.1016/j.jbi.2019.103208

Hastings, H. (1915). Syphilis of the internal ear (hereditary). *Journal of the American Medical Association*, *LXV*(7), 607-608. https://doi.org/10.1001/jama.1915.02580070041011

Hughes, K. B., & Murray, D. P. J. (1981). Congenital syphilis presenting as a case of deafness. *Journal of Laryngology and Otology*, *95*(1), 75-80. https://doi.org/http://dx.doi.org/10.1017/s0022215100090435

Indesteege, F., & Verstraete, W. L. (1989). Meniere's disease as a late manifestation of congenital syphilis. *Acta oto-rhino-laryngologica Belgica*, *43*(4), 327-333.

Ittkin, P., & Tamari, M. J. (1953). Studies of syphilis on the ear and penicillin. *The American journal of the medical sciences*, *225*(4), 427-445.

Joanna Briggs, I. (2017). *Critical Appraisal Checklist for Case Reports*.

Joint Committee on Infant Hearing, T. (2007). Year 2007 Position Statement: Principles and Guidelines for Early Hearing Detection and Intervention Programs. *Pediatrics*, *120*(4), 898-921. https://doi.org/10.1542/peds.2007-2333
Joint Committee on Infant Hearing, T. (2019). Year 2019 Position Statement: Principles and Guidelines for Early Hearing Detection and Intervention Programs. *Journal of Early Hearing Detection and Intervention*, *4*(2).
Kameswaran, S., Rajenderkumar, P. V., Jacob, K. T., & Manoharan, S. (1976). Congenital hearing loss in venereal disease. *Indian Journal of Otolaryngology*, *28*(3), 98-98. https://doi.org/10.1007/BF02990972
Karmody, C. S., & Schuknecht, H. F. (1966). Deafness in congenital syphilis. *Archives of otolaryngology (Chicago, Ill. : 1960)*, *83*(1), 18-27.
Keidel, A., & Kemp, J. E. (1923). Deafness in late congenital syphilis. *Southern Medical Journal*, *16*(9), 647-651. https://doi.org/10.1097/00007611-192309000-00001
Kerr, A. G., & Adams, D. A. (1983). Congenital syphilitic deafness - a long-term follow-up. *Advances in oto-rhino-laryngology*, *31*(2o7, 0242534), 247-252.
Kerr, A. G., Smyth, G. D., & Cinnamond, M. J. (1973). Congenital syphilitic deafness. *The Journal of laryngology and otology*, *87*(1), 1-12.
Kerr, A. G., Smyth, G. D., & Landau, H. D. (1970). Congenital syphilitic labyrinthitis. *Archives of otolaryngology (Chicago, Ill. : 1960)*, *91*(5), 474-478.
Khetarpal, S., Kempf, E., & Mostow, E. (2011). Congenital Syphilis: Early- and Late-Stage Findings of Rhagades and Dental Anomalies. *Pediatric Dermatology*, *28*(4), 401-403. https://doi.org/https://doi.org/10.1111/j.1525-1470.2010.01288.x
Kivekas, I., Vasama, J.-P., & Hakomaki, J. (2014). Bilateral temporal bone otosyphilis. *Otology & neurotology : official publication of the American Otological Society, American Neurotology Society [and] European Academy of Otology and Neurotology*, *35*(2), e90-91. https://doi.org/https://dx.doi.org/10.1097/MAO.0b013e3182a3603f
Lim, J., Yoon, S. J., Shin, J. E., Han, J. H., Lee, S. M., Eun, H. S., . . . Park, K. I. (2021). Outcomes of infants born to pregnant women with syphilis: a nationwide study in Korea. *BMC Pediatrics*, *21*(1), 47-47. https://doi.org/10.1186/s12887-021-02502-9
Lima, P. T., Goldbach, M. G., Monteiro, M. C., & Ribeiro, M. G. (2015). A triagem auditiva neonatal na Rede Municipal do Rio de Janeiro, Brasil. *Ciência & Saúde Coletiva*, *20*(1), 57-63. https://doi.org/10.1590/1413-81232014201.21002013
Moola S, M. Z., Tufanaru C, Aromataris E, Sears K, Sfetcu R, Currie M, Qureshi R, Mattis P, Lisy K, Mu P-F. (2020). Chapter 7: Systematic reviews of etiology and risk. In M. Z. Aromataris E (Ed.), *JBI Manual for Evidence Synthesis*. Joanna Briggs Institute.
Morton, R. S. (1955). Late Congenital Syphilitic Nerve Deafness. *Sexually Transmitted Infections*, *31*(4), 242-244. https://doi.org/10.1136/sti.31.4.242
Morton, R. S. (1957). Cortisone Therapy in Congenital Syphilitic Nerve Deafness. *The Journal of Laryngology & Otology*, *71*(12), 850-852. https://doi.org/10.1017/S0022215100052567
Munn Z, M. S., Lisy K, Riitano D, Tufanaru C. (2020). Systematic reviews of prevalence and incidence.
Murphy, E. A., Hahn, R. D., & Haskins, H. L. (1958). NERVE DEAFNESS ASSOCIATED WITH CONGENITAL SYPHILIS: REPORT OF A CASE TREATED WITH PREDNISONE AND PENICILLIN. *Journal of the American Medical Association*, *167*(16), 1925-1928. https://doi.org/10.1001/jama.1958.72990330001006

Onoda, R. M., Azevedo, M. F. d., & Santos, A. M. N. d. (2011). Triagem auditiva neonatal: ocorrência de falhas, perdas auditivas e indicadores de riscos. *Brazilian Journal of Otorhinolaryngology*, *77*(6), 775-783. https://doi.org/10.1590/S1808-86942011000600015

Page, M. J., McKenzie, J. E., Bossuyt, P. M., Boutron, I., Hoffmann, T. C., Mulrow, C. D., . . . Moher, D. (2021). The PRISMA 2020 statement: an updated guideline for reporting systematic reviews. *Bmj*, *372*, n71. https://doi.org/10.1136/bmj.n71

Patterson, M. E. (1967). Congenital luetic hearing impairment. Treatment with prednisone. *Transactions of the Pacific Coast Oto-Ophthalmological Society annual meeting*, *51*(war, 7506126), 235-246.

Perlman, H. B., & Leek, J. H. (1952). Late congenital syphilis of the ear. *The Laryngoscope*, *62*(11), 1175-1195. https://doi.org/10.1288/00005537-195211000-00003

Pessoa, L., & Galvao, V. (2011). Clinical aspects of congenital syphilis with Hutchinson's triad. *BMJ case reports*, *2011*(101526291). https://doi.org/https://dx.doi.org/10.1136/bcr.11.2011.5130

Public Health Agency of Canada, T. (2014). *The Chief Public Health Officer's Report on the State of Public Health in Canada*. G. o. Canada.

Public Health Agency of Canada, T. (2022). Infectious syphilis and congenital syphilis in Canada, 2021. *Canada Communicable Disease Report (CCDR)*, *48*(11/12), 587.

Simms, I., Tookey, P. A., Goh, B. T., Lyall, H., Evans, B., Townsend, C. L., . . . Ison, C. (2017). The incidence of congenital syphilis in the United Kingdom: February 2010 to January 2015. *BJOG: An International Journal of Obstetrics & Gynaecology*, *124*(1), 72-77. https://doi.org/10.1111/1471-0528.13950

Singhal, P., Patel, P., & Marfatia, Y. S. (2011). A case of congenital syphilis with Hutchinson's triad. *Indian Journal of Sexually Transmitted Diseases*, *32*(1), 34-36. https://doi.org/http://dx.doi.org/10.4103/0253-7184.81252

Smyth, G. D. L., Kerr, A. G., & Cinnamond, M. J. (1976). Deafness due to syphilis. *Journal of the Otolaryngological Society of Australia*, *4*(1), 36-40.

Whiting, P., Savović, J., Higgins, J. P., Caldwell, D. M., Reeves, B. C., Shea, B., . . . Churchill, R. (2016). ROBIS: A new tool to assess risk of bias in systematic reviews was developed. *J Clin Epidemiol*, *69*, 225-234. https://doi.org/10.1016/j.jclinepi.2015.06.005

Wilson, W. R., & Zoller, M. (1981). Electronystagmography in congenital and acquired syphilitic otitis. *The Annals of otology, rhinology, and laryngology*, *90*(1 Pt 1), 21-24.

Wright, I. (1968). Late-onset hearing loss due to congenital syphilis. *International Journal of Audiology*, *7*(3), 302-310. https://doi.org/10.3109/05384916809074336

Yearsley, M. (1910). Cases of Acquired Deaf-Mutism due to Congenital Syphilis. *The Journal of Laryngology, Rhinology, and Otology*, *25*(5), 234-239. https://doi.org/10.1017/S1755146300184756

Zoller, M., Wilson, W. R., & Nadol, J. B., Jr. (1979). Treatment of syphilitic hearing loss. Combined penicillin and steroid therapy in 29 patients. *The Annals of otology, rhinology, and laryngology*, *88*(2 Pt 1), 160-165.

Figure Legend

PRISMA flow diagram

Table 1 – Quality assessment of studies

Type of study	Author (Year) Country	Quality Assessment	Comments
Children with HL were screened for CS	Gururaj (1900) India	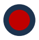	-Quota Sampling<br>-Descriptive statistics<br>- Method of diagnosis of CS or HL not described
	Yearsley (1910) UK	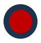	-Only the demographics and phenotypes of patients in case reports described<br>-Method of diagnosis of CS or HL not described<br>- Inclusion or exclusion criteria not clear<br>-Longitudinal reporting and follow up not defined
	Wright (1968) UK	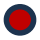	-Quota Sampling<br>-Descriptive statistics - no correlation between CS and HL proven<br>--Method of diagnosis of CS or HL not described
	Kameswaran (1976) India	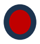	-Judgmental non-probability sample<br>-Method of diagnosis of HL subjective [audiometry]<br>-Descriptive statistics - no correlation between CS and HL proven
	Ganga (1990) India	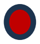	-Judgmental sampling<br>-Descriptive statistics - no correlation between CS and HL proven<br>-Main objective was to look at the psychological impact rather than the etiology of HL
Children with CS were screened for HL	Keidel (1923) US	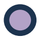	-Adequate sample size<br>-Setting described<br>-Descriptive study with no data analysis
	Ittkin (1953) US and Canada	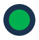	-Adequate sample size<br>-Study subjects and setting described<br>- Sufficient data with statistical analysis<br>- Identification of the condition valid
	Gleich (1994) US	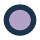	-A quota sample used<br>- ABR used for auditory screening<br>-Descriptive study<br>-Correlation could not be proved
	Simms (2016) UK	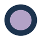	-A quota sample was used<br>-Definition of CS precise but no clear definition of HL
	Lim (2021) South Korea	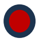	- A quota sample used<br>- No clear diagnostic criteria or tools for HL<br>-Though they applied statistics, not clear what they were trying to prove
	Besen (2022) Brazil	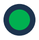	-Adequate sample size<br>-Study subjects and setting described<br>-Sufficient data and analysis<br>- Fails to describe how CS diagnosed

	Aguiar (2022) Brazil		- Adequate sample size<br>-Study subjects and setting described<br>- ABR used for auditory screening. Note that equipment was changed during study but no statistical significance.<br>- Sufficient data and statistical analysis<br>-Identification of condition valid
Case series	Yearsley (1910) UK		-Only the demographics and phenotypes of patients in case reports were described.<br>- Method of diagnosis of CS or HL not described<br>- Inclusion or exclusion criteria unclear<br>-Longitudinal reporting and follow up not defined
	Fraser (1916) UK	.	-Patients demographics and phenotypes were described.<br>-No diagnostic tests used to confirm HL
	Perlman (1952) US		-Demographics, and audio-vestibular loss described.
	Morton (1955) UK		-Demographics, CS diagnosis were described.<br>-Audio vestibular symptoms depicted more than diagnostics.
	Morton (1957) UK		-Brief demographics and symptomatology<br>- Audio-vestibular diagnosis not reported<br>- Method of CS diagnosis not described
	Baron (1967) France		-No patient's demographics, patient's history, clinical condition, or invention described<br>- only the diagnostic tests or assessment methods in addition to intervention and post intervention clinical conditions<br>-Adverse effects and objectives not clear.
	Dawkins (1968) UK		-Demographics and diagnostics well described
	Patterson (1968) US		-Demographics and diagnostics well described
	Kerr (1970) Ireland		-Brief demographics<br>- Methods of CS diagnosis described
	Indesteege (1989) Belgium		-Brief demographics<br>- Audio vestibular diagnosis of Leutic Meniere's disease explained<br>-Method of CS diagnosis explained
Case reports	Hastings (1915) US		-Demographic described well<br>-Audio-vestibular finding explained<br>- Longitudinal results of treatment assessed
	Murphy (1958)		-Demographic described well<br>-Audio-vestibular finding explained

	US		-Longitudinal results of treatment assessed
	Karmody (1966) US		-Demographic described well<br>--Audio-vestibular finding explained<br>-Histopathology added when autopsy performed
	Hughes (1981) Germany		-Demographic described well<br>-Audio-vestibular finding explained<br>-Longitudinal results documented
	Khetarpal (2011) US		-Demographic described<br>-More emphasis on other manifestation than on HL
	Pessoa (2011) Brazil		-Demographics described<br>-Symptoms and signs of CS explained including HL<br>-Not all hearing assessments included
	Singhal (2011) India		-Method of diagnosis of CS described<br>-Also had a cleft palate that could have contributed to HL.
	Kivekas (2014) US		-Demographics described. This is a case report of radiological findings in the temporal bones because of congenital syphilis.<br>-Symptoms and signs were explained as well as interventions in addition to radiologic findings.
Studies of an intervention	Hahn (1961) US		- Small number/ cohort<br>-Confounding factors were not accounted for<br>-It was mentioned that longitudinal results are difficult to obtain
	Kerr (1973) Ireland		-Patients lost to follow up not accounted for<br>-Longitudinal follow up unclear
	Smyth (1976) Ireland		- Exclusion criteria mentioned.
	Zoller (1979) US		-Detailed description of demographics and clinical picture<br>-Audiometric results before and after treatment provided<br>-No group comparison
	Wilson (1981) US		-Confounding factors not accounted for<br>-Longitudinal results unclear
	Dobbin (1983) US		-Stats purely descriptive<br>-No mention of confounding factors
	Kerr (1983) Ireland		-Adverse effects mentioned<br>-Conclusion articulated

Legend: CS – congenital syphilis; HL – hearing loss; UK – United Kingdom; US – United States

High risk of bias

Unclear or moderate risk of bias

Low risk of bias

Table 2 – Studies where multiple children with hearing loss were screened for congenital syphilis

**Author (Year) Country**	**Description of participants screened for CS**	**Number of children with CS**
Gururaj (1900) India	300 children with HL at a school for children with visual and hearing impairment	15 (5%)
Yearsley (1910) UK	225 children with acquired HL in “deaf centres” in London with acquired HL (did not test 229 with congenital HL or 46 with unclear timing of onset of HL)	17 (8%)
Wright (1968) UK	1157 patients with SNHL at the Royal National Throat, Nose, and Ear Hospital 1963 to 1966	20 (2%)
Kameswaran (1976) India	367 children at schools for the hearing impaired	1 (0.3%)
Ganga (1990) India	100 children at schools for the hearing impaired	1 (1%)

Legend: CS – congenital syphilis; HL – hearing loss; SNHL – sensorineural hearing loss

Table 3 – Studies where children with congenital syphilis were screened for hearing loss

**Author (Year) Country**	**Population screened**	**Participants with HL**	**Hearing loss pattern**
Keidel (1923) US	230 with CS seen at Syphilis Clinic at John Hopkins Hospital 1917-1923.	23 (10%)	Late SNHL was found in 22 patients plus one had deafness due to gummatous condition in nasopharynx. Onset occurred from 7 to 27 years of age. Four had sudden HL (over the course of a week.). Seven had bilateral HL.
Ittkin (1953) US and Canada	59 with late CS	11 (19%)	Two had sudden onset HL, 3 had gradual onset, with no comment on the other 6.<br><br>Three had excessive high tone HL and 8 had subnormal HL (defined as narrowed range to voice, forks and audiometric tests with impaired bone conduction
Gleich (1994) US	75 neonates with positive FTA-ABS or MHA-TP and Apgar > 9 at 5 minutes of life (provide no data on how many had CS versus passive antibodies – 41 had CSF obtained and none had evidence for CNS syphilis)	0	NR
Simms (2016) UK	17 patients born 2010 -2015 with possible or proven CS	1 (6%)	NR
Lim (2021) South Korea	250 neonates treated for possible or proven CS	34 (14%) including 6 of 14 (43%) with neurosyphilis	~~NR~~ 14% had hearing impairment according to The International Classification of Diseases-10 codes<br>spelling error?
Besen (2022) Brazil	21,434 newborns evaluated in a Brazilian reference hearing health service of the UNHS Program.	22 (6%)	NR
Aguiar (2022) Brazil	93 newborns divided into three groups.<br>Group 1: Prenatal treatment for syphilis (n=7)<br>Group 2: Treatment for syphilis after birth (n=37)<br>Group 3: Control group with no syphilis (=49)	Group 1: 0/7<br>Group 2: 2/37 (5%)<br>Group 3: 3/49 (6%)	NR

Legend: CSF – cerebrospinal fluid; CNS – central nervous system; CS – congenital syphilis; HL – hearing loss; NR – not reported; SNHL – sensorineural hearing loss

Table 4A - Case series of hearing loss attributed to congenital syphilis

**Author (Year) Country**	**Demographics**	**Hearing loss assessment**	**Interventions**
Yearsley (1910) UK	17 cases with onset at 6 to 14 years (unknown for 2 cases).<br>All had interstitial keratitis (preceded deafness in at least 10 cases).	Bone conduction, otoscopy, traditional ear exam -used subjective unconfirmed behavioral measures.	NR
Fraser (1916) UK	33 cases between the ages of 7 and 38 with HL recognition at 3,6,7 (n=2), 8 (n=4), 9 (n=2), 10 (n=2), 11 (n=3),12,14, 15 (n=2) , 16, 17, 18, 19 (n=2), 20, 24, 25, 27 (n=2), 21(n=2), 30, and 33 years.<br>HL sudden (N=8), gradual (N=14) and unknown (N=11).<br>All but one had interstitial keratitis.	Bone conduction, otoscopy, vestibular irritation test.	One case received pot<br>pilocarpine injection v
Perlman (1952) US	11 cases with recognition of HL loss at 9, 11, 12, 17 (N=2), 18, 19, 23, 33, and 35 (N=2) years.<br>All had interstitial keratitis.	All but one had bilateral HL. Cold caloric test abnormal in 10 of 11 and fistula test positive in 2 of 9 where results reported.	Bismuth, arsenic +/- p<br>improvement – all 5 th<br>improvement
Morton (1955) UK	4 cases with onset at approximately ages 15, 34, 35 and 53 years	Traditional audiometry inconsistently mentioned	All received beta-pyri<br>penicillin – 3 reported
Morton (1957) UK	4 cases with onset at approximately 18, 24 and 35 years (unknown for one patient)	Unclear	-Penicillin treatment i<br>prednisone 10 mg thre<br>by 5 mg twice daily fo<br>subjective improveme
Baron (1967) France	3 children aged 10, 11 and 16 years with maternal diagnosis made after diagnosis in child	Traditional audiometry: moderate bilateral HL (N=1), minimal bilateral hearing loss (N=1), mild HL on right and minimal on left	Case 1: Penicillin 80 r<br>Deterioration of hearin<br>course antibiotics. Imp<br>Case 3: Removal of ve<br>tubal massages, and an<br>hearing on the left and
Dawkins (1968) UK	5 cases with onset of HL at age 13, 20, 42, 37 and 55 years	Traditional audiometry: Moderate HL in right ear and moderate-severe in left (N=1), minimal HL in right ear and mild HL in left (N=1), mild HL bilaterally (N=1),<br>profound HL in right and moderate HL in left (n=1) and mild HL in right and severe in left (N=1)	All treated with predn<br>Case 1 right ear impro<br>but only to pure tone a<br>moderate improvemen
Patterson (1968) US	4 cases with onset of HL at age 26,32 29, and 39 years)	Limited results reported	All received predniso<br>improvement in symp<br>improved speech discr<br>maintenance predniso
Kerr (1970) Ireland	3 cases aged 46 to 55 years with onset of HL at ages 26, 47 and 53 years	Pure tone threshold and speech discrimination: Left ear HL followed by right ear HL with vertigo (N=1), fluctuating HL with rotational vertigo (N=1) and bilateral HL (N=1)	All cases: high dose p<br>prednisone for ten day<br>to have improved spee<br>ear and vertigo improv

Indesteege (1989) Belgium	5 cases aged 50 to 61 years at time of study (not clear when HL started)	Audiometry, tympanometry and stapedial reflex. Found to have symmetrical HL with Meniere’s disease and tinnitus (N=3), mixed, bilateral perceptive, symmetric, and fluctuant HL with Meniere’s disease and tinnitus with vertigo (N=1) and bilateral perceptive and fluctuant HL with Meniere’s disease, tinnitus and vertigo	2 cases treated with co[illegible] due to side-effects

Legend: CSF – cerebrospinal fluid; CNS -central nervous system; HL – hearing loss; NR – not reported; UK – United Kingdom: US – United States

Table 4B - Case reports of hearing loss attributed to congenital syphilis

**Author (Year) Country**	**Patient demographics**	**Diagnosis of congenital syphilis**	**Hearing loss assessment**	**Interventions**
Hastings (1915) US	20-year-old female with Hutchinson's triad	Maternal diagnosis prenatally, but asymptomatic for 19 years.	Traditional audiometry	Mercurial topical treatment
Murphy (1958) US	20-year-old female with HL diagnosed at age 9 years	Age 9 years	Traditional audiometry with mild mixed HL, more marked in the right ear.	Multiple treatments with penicillin and prednisone
Karmody (1966) US	44-year-old male with bilateral HL diagnosed at age 18	Maternal diagnosis after birth	Traditional audiometry with profound HL in right ear and severe HL in left ear	Penicillin at age 32 and then at age 44 with no improvement
Hughes (1981) Germany	39-year-old male with bilateral HL fluctuating on the right	Unreported	Traditional audiometry	Penicillin 500000 U, probenecid, and prednisolone
Khetarpal (2011) US	5-year-old with bilateral hearing loss with early and late features of CS	4 months – ruled out neurosyphilis	No reported hearing assessment	Penicillin at 10 months of age
Pessoa (2011) Brazil	7-year-old female with Hutchison's triad	2 years	NR	Penicillin – unknown dose
Singhal (2011) India	14-year-old female with Hutchison's triad	14 years	NR	Benzathine penicillin 2.4 million units intramuscularly
Kivekas (2014) US	75-year-old female with bilateral hearing loss since age 10	Positive syphilis diagnosis after chronic interstitial keratitis at unknown age – in 1950s	No reported hearing assessment. 76% bisyllabic work scores with right ear cochlear implant.	None

Legend: HL – hearing loss; NR – not reported

Table 5 – Studies with an intervention for hearing loss that was attributed to congenital syphilis with minimal details on individual patients

**Author (Year)**	**Total participants**	**Intervention**	**HL characteristics prior to intervention**	**HL characteristics fo**

Country				
Hahn (1961) US	Group A: Patients with syphilitic neural deafness (N=19)<br>Group B: Patients with non-syphilitic neural deafness (N=20)	Prednisone 30 mg daily in divided doses for one week, then 20 mg daily, then 2.5 mg taper per day each month if progressing well. Six patients received penicillin at the start of prednisone therapy.	Group A: On average, lower discrimination scores prior to treatment.<br>Group B: Cases described often had sudden HL with decreases in discrimination scores noted.	Group A: Definite im[illegible] discrimination score [illegible] patients. Two patient[illegible] discrimination scores[illegible] saw no significant im[illegible]<br>Group B: Very slight [illegible] cases, but overall, no [illegible]
Kerr (1973) Ireland	12	All got ampicillin, at least 3 also got prednisone, 4 of whom also got ACTH.	Case 1: Fluctuations in pure tone hearing and speech discrimination for 2-3 years prior to treatment.<br>Case 2 and 3: Profound deafness for greater than 6 months with speech discrimination score of 0.<br>Case 4: High speech discrimination score, but low pure tone hearing score with sudden drop in speech discrimination prior to treatment.	All had improved spe[illegible]
Smyth (1976) Ireland	19	Group A (N=9): Ampicillin for 4 weeks.<br>Group B (N=13 – includes 3 who failed from Group A): ampicillin plus prednisone 10 mg TID for 10 days (if no improvement additional 10 days of prednisone at same dose).<br>Group C (N=8, all of whom failed in Group B): ampicillin, prednisone and ACTH 40 to 120 units per week (duration of treatment not specified)	Group A: 6 ears initially had low speech discrimination scores; 9 ears had satisfactory hearing.<br>Group B: 11 ears had profoundly HL and 12 had significant HL.<br>Group C: 6 ears with profound and 8 ears with significant HL.<br>Overall: 2 patients had severe tinnitus and 5 patients had vertigo.	Group A: 6 ears whic[illegible] speech discriminatio[illegible] 20% in speech discri[illegible] only maintained in 2 [illegible] satisfactory hearing a[illegible] hearing.<br>Group B: All initially [illegible] relapsed.<br>Group C: All improv[illegible] All but one had servi[illegible] follow-up of maximu[illegible]
Zoller (1979) US	12	Penicillin G plus 80 mg prednisone every other day	Mean SRT in left ear was 54.5 dB and 51.0 dB on the right.<br>Mean speech discrimination score was 47.2% on the left and 61.6% on the right	3 patients had sustain[illegible] at 1 year, 1 had trans[illegible] 5 had no improveme[illegible]

Wilson (1981) US	7	3 months of weekly benzathine penicillin G and 80 mg of prednisone orally every other day up to one month.	All had vertigo	Vertigo improved in 4
Dobbin (1983) US	13	Penicillin G IV daily for 2 weeks followed by benzathine penicillin every other week for 10 weeks. Prednisone was co-administered starting at 80 mg every other morning for 1 month and tapered based on patient response.	Unknown	9 of 26 ears showed a<br>2 ears showed improv<br>threshold while 8 ears<br>improvements (one ea<br>improvements in both<br>transitory improveme<br>Only 4 ears had any la<br>speech discrimination<br>threshold.
Kerr (1983) Ireland	17	Group A (N=1): Ampicillin for 4 weeks<br>Group B (N=7): Ampicillin and prednisone 10 mg for 10 days with 10-day taper.<br>Group C (N=6): Ampicillin, prednisone, and ACTH (40 units to 120 units) for prolonged course<br>Group D (N=3) – deceased<br>Followed for mean 10.5 years	Group A: Fluctuating HL.<br>Group B: Mean speech discrimination score 62%. 4 ears had profound, 6 had fluctuating HL and 4 were stable.<br>Group C: Mean speech discrimination score 46%. 3 ears had profound and 8 had fluctuating HL.	Group A: Fluctuating<br>discrimination score n<br>Group B: No response<br>profound HL. 4 of 6 e<br>HL remained fluctuat<br>speech discrimination<br>stable and 1 ear had a<br>speech discrimination<br>56%. For the 4 stable<br>2 remained stable, 1 d<br>and 1 developed fluct<br>treatment.<br>Group C: No response<br>profound HL. Out of t<br>HL, 1 remained stable<br>profound HL. The oth<br>fluctuating hearing lo

Legend: ACTH - Adrenocorticotropic hormone; HL – Hearing loss; PTA – pure tone average; SRT – speech recognition threshold

PRISMA flowchart

Click here to access/download;Figure;PRISMA Flowchart Final - Sept 28.docx

2021 Hafeez/Gomaa Congenital Syphillis and Hearing Loss

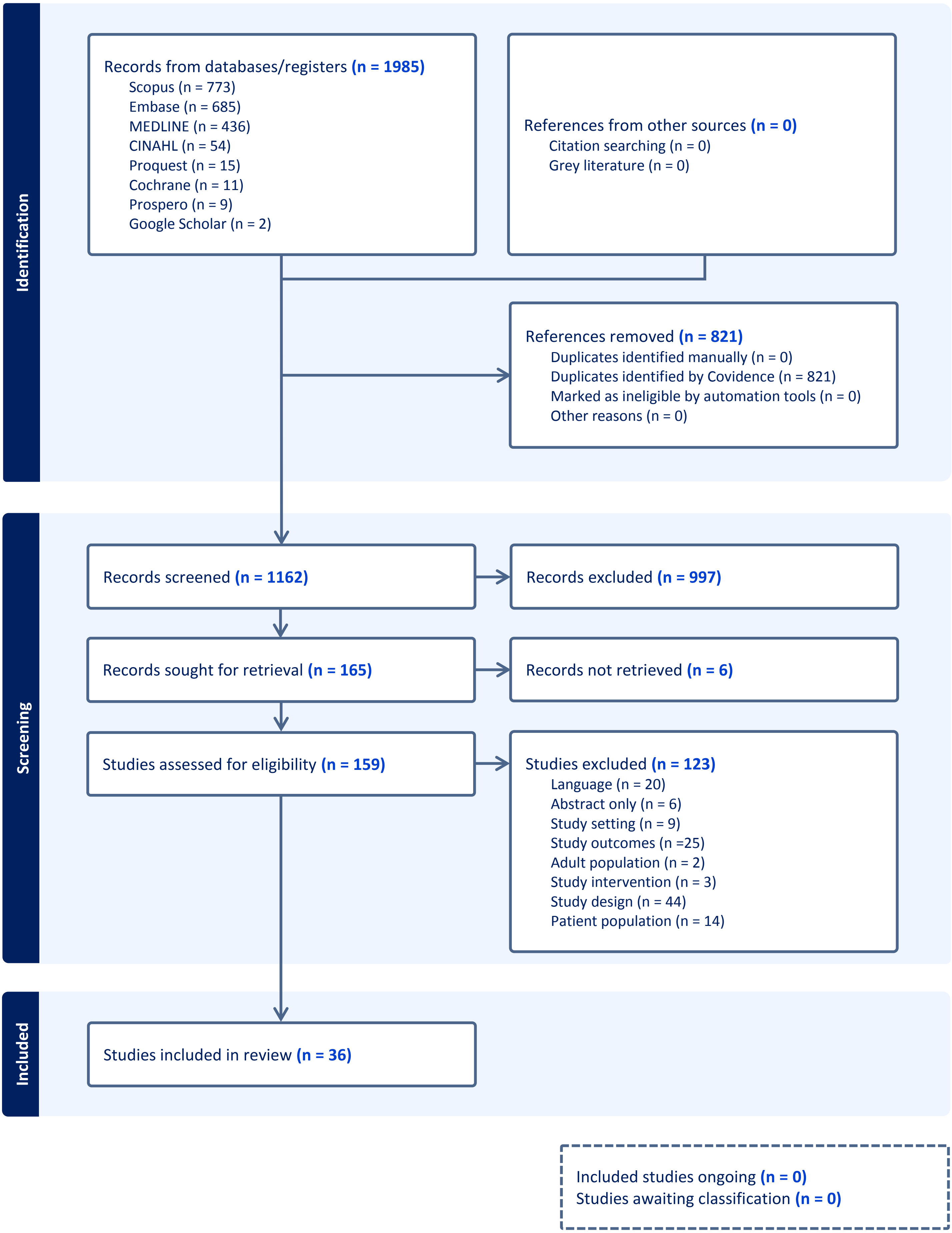

3rd August 2023

covidence

Click here to access/download
Supporting Information
PRISMA-ScR-Fillable-Checklist_10Sept2019.docx

Appendix A - Search strategy

Click here to access/download
Supporting Information
Appendix A - scoping review - congenital syphilis (1).docx

Click here to access/download
Supporting Information
JBI Critical Appraisal Checklist_all studies Sept 28.docx
