# Supplementary material for: Scoping review of hearing loss attributed to congenital syphilis
Source: PLoS One. 2024 Apr 26;19(4):e0302452. doi: 10.1371/journal.pone.0302452 (PMC11051613; doi:10.1371/journal.pone.0302452)
Supplement: Attachment — Submitted filename: Response to reviewers Dec 11.docx [file pone.0302452.s005.docx]

Dear Editor,

Thank-you for agreeing to review a revision of our manuscript. We have addressed the comments of the reviewers as follows:

A. **Editor’s Comments:**

The authors have put a lot of effort into this article. But the manuscript is not conveying the important message of this scoping review. A lot of key information is missing in the introduction, results, and the discussion section.
1. What is the purpose of this scoping review?

**Response: The submitted version of the manuscript stated in the introduction: “There are no published data on the incidence or characteristics of hearing loss from CS. We therefore performed a scoping review to better characterize this entity. This will inform the studies that need to be done to determine the incidence and age of onset of hearing loss from CS, the severity of hearing loss, and interventions that warrant further study”.**

**To make our objectives clearer, we added the following to this paragraph:**

**Our specific questions were:**

- **How often is hearing loss due to congenital syphilis?**
- **What is the incidence of hearing loss in children with congenital syphilis?**
- **When hearing loss occurs from congenital syphilis, what is the usual age of onset? Is it unilateral or bilateral? How severe is it? How rapidly does it progress?**
- **Is there evidence for any interventions for treatment of hearing loss attributed to congenital syphilis?**

**We also added a sentence to make it clearer that there are no guidelines for ongoing management of hearing loss with CS.**

2. What are the authors trying to convey from this scoping review? Do the authors think that there should be a systematic review conducted based on a scoping review?

**Response: We agree that we should have made it clearer that the scoping review showed a great need for higher quality studies. We have added the following sentence to the first paragraph in the discussion: “****One cannot determine the incidence or characteristics of hearing loss from CS or the efficacy of interventions from this review. It seems unlikely that a systematic review would yield answers to any of these questions.”**

**3.** How does congenital syphilis cause hearing loss? Hearing loss is the sequelae? or medicine given to treat syphilis causes hearing loss?

**Response: We agree that we could have explained this better and added to the first paragraph: “The Centers for Disease Control and Prevention state: “Otosyphilis is caused by an infection of the cochleovestibular system with T. pallidum and typically presents with sensorineural hearing loss, tinnitus, or vertigo. Hearing loss can be unilateral or bilateral, have a sudden onset, and progress rapidly.” Penicillin is the only treatment used and it is not known to be an ototoxic drug. We now state “Almost all cases of CS are treated with penicillin which is not known to be ototoxic.”**

4. What is the current gap in the knowledge base on congenital syphilis?

**Response: As noted above, the introduction of the submitted version of the manuscript stated: “There are no published data on the incidence or characteristics of hearing loss from CS.”**

5. What is the available evidence in individuals with congenital syphilis?

**Response: It is not clear what the editor is asking. Our scoping review summarizes the evidence on hearing loss. Did they want us to talk about all aspects of congenital syphilis?**

6. I did not see the overview or the map of evidence on congenital syphilis.

**Response: We used the Cochrane methods for our scoping review (See** [**Scoping reviews: what they are and how you can do them | Cochrane Training**](https://training.cochrane.org/resource/scoping-reviews-what-they-are-and-how-you-can-do-them) **). I do not think that an overview or a map of evidence are a mandatory part of a scoping review. A review article on evidence mapping (Miake-Lye IM et al. What is an evidence map? A systematic review of published evidence maps and their definitions, methods, and products. Syst Rev. 2016 Feb 10:5:28. doi: 10.1186/s13643-016-0204-x) states: “… scoping reviews include “a descriptive narrative summary of the results” whereas evidence maps identify evidence gaps, and both use a tabular format to depict a summary of literature characteristics “. Given the lack of evidence we uncovered, it would be very difficult to make a useful evidence map.**

7. How was the hearing research conducted in the past and currently?

**Response: We are sorry but we could not determine what the editor is asking. We tell the reader that all but one study were observational studies so there is not a specific methodology.**

**B. Reviewer’s Comments:**

Overall Evaluation:
The paper offers valuable insights into hearing loss attributed to congenital syphilis. The authors put a lot of effort into finding the relevant literature and conducting the scoping review. Overall, I find the work satisfactory. However, I find changes are required to the text. There were a few spelling and grammar mistakes. I recommend improving the organization of the content in the paper.

Major Contributions:
Relevance of Topic: The topic is of high importance and is aptly chosen.

Comprehensiveness: The review covers a wide array of studies and provides a comprehensive overview.

Originality: The topic that the authors selected is a novel research area. There is adequate information; however, I suggest improvements to further identify gaps and mention potential areas for future research.

Areas for improvement:
A document with suggestions for improvements is attached. Please refer to the document. The document also contains the paper with corrections and questions marked in red. Please refer.

Summary and Recommendation:
Overall, this manuscript is a commendable effort in synthesizing the current knowledge on hearing loss attributed to congenital syphilis. It provides an insightful overview, though some areas require refinement for clarity and depth. With revisions, particularly in the introduction, methodology, and discussion sections, this manuscript will be a valuable addition to the literature. I recommend acceptance of the manuscript after revisions.

1. An explicit statement of research questions and objectives is not provided. I recommend rethinking and clearly rewriting the text. Please use bullet points and proper formatting. It helps the reader to understand clearly.

**Response: Thanks for pointing out that our objectives were not as clear as we thought that they were. We added the following to the introduction:**

**Our specific questions were:**

- **How often is hearing loss due to congenital syphilis?**
- **What is the incidence of hearing loss in children with congenital syphilis?**
- **When hearing loss occurs from congenital syphilis, what is the usual age of onset? Is it unilateral or bilateral? How severe is it? How rapidly does it progress?**
- **Is there evidence for any interventions for treatment of hearing loss attributed to congenital syphilis?**

**2.** State the process for selecting sources of evidence (i.e., screening and eligibility) included in the scoping review. Reviewer says: The selection process is clear. However, I recommend re-think and clearly describe in the text. Use bullet points and proper formatting. Formatting issues were identified.

**Response: I am sorry but it was not clear what the reviewer wanted us to change. It is not common to use bullet points when describing the sources that were searched for a systematic or scoping review.**

**3.** For each source of evidence, present characteristics for which data were charted and provide the citations. Reviewer states: Charts are included. I recommend moving the charts below to relevant sections in the text. Otherwise, it was challenging to scroll down and go back to the text to relate what the authors are trying to describe.

**Response: I apologize for this error. I clearly did not read the author instructions carefully. The tables have been moved.**

**4.** Discussion - Found complex sentence structures. Look into the text again and think of clear explanation.

**Response: We tried to make the text clearer throughout the discussion.**

5. The reviewer asked that we tell the reader the number of articles included in the “Design” section of the abstract.

**Response: It is customary not to divulge results until the “Results” section of the abstract.**

**6.** The reviewer suggests adding a reference to the sentence “Syphilis is a sexually transmitted infection caused by the bacterium Treponema pallidum.”

**Response: It seems unlikely that the reader would require a reference to believe that this statement is correct. We can add one if required.**

7. We were asked to double-check whether the statistics that we quote in the introduction from previous studies are correct.

**Response: We have no way of verifying this but have quoted the studies accurately.**

8. Methods – We were asked where the keywords are.

**Response: As far as we can tell, PLOS ONE does not request keywords. We can add some if required.**

**9.** Articles published in English were screened by two reviewers independently [AH, KC], and conflicts were resolved by a senior author [JR, NG]. Articles published in French had a single reviewer [FS]. There were no Portuguese articles. Because of the small number of recent articles, preprints were included. The protocol has not been published.´The reviewer stated that perhaps we did not need to include this information and asked whether the protocol was in Prospero.

**Response: We can omit this information if requested. The protocol is not in Prospero as unfortunately they only publish protocls for systematic reviews – not scoping reviews.**

10. Figure 1 is far away from the text.

**Response: Instructions to authors request that figures be submitted as a separate file.**

**11. “** One study from the modern era showed an incidence of 6% (22/342) (Besen et al., 2022).” Reviewer states: “not required or explain in words”, I think referring to 22/342.

**Response; We think that it is helpful for the reader to not have to look at the table to determine the sample size for this statistic, but can omit that if requested.**

**12. Multiple minor changes were made in the text as requested.**
