# Supplementary material for: Scoping review of hearing loss attributed to congenital syphilis
Source: PLoS One. 2024 Apr 26;19(4):e0302452. doi: 10.1371/journal.pone.0302452 (PMC11051613; doi:10.1371/journal.pone.0302452)
Supplement: Attachment — Submitted filename: Response to reviewers - hearing loss.docx [file pone.0302452.s006.docx]

Thanks for agreeing to review a revised version of our manuscript. We have addressed the suggestions as follows:

Please ensure that your manuscript meets PLOS ONE's style
requirements, including those for file naming. The PLOS ONE style templates can
be found at


<https://journals.plos.org/plosone/s/file?id=wjVg/PLOSOne_formatting_sample_main_body.pdf>
and


<https://journals.plos.org/plosone/s/file?id=ba62/PLOSOne_formatting_sample_title_authors_affiliations.pdf>.

Response: *Changes were made to meet these requirements.*

2. We note that your Data Availability Statement is
currently as follows: This is a systematic review so all data can be derived from the included manuscripts.


Please confirm at this time whether or not your submission
contains all raw data required to replicate the results of your study. Authors
must share the “minimal data set” for their submission. PLOS defines the
minimal data set to consist of the data required to replicate all study
findings reported in the article, as well as related metadata and methods
(<https://journals.plos.org/plosone/s/data-availability#loc-minimal-data-set-definition>).

Response: The manuscript contains all raw data required to replicate the results of the study.

3. Please review your reference list to ensure that it is complete and correct. If you have cited papers that have been retracted, please include the rationale for doing so in the manuscript text, or remove these references and replace them with relevant current references. Any changes to the reference list should be mentioned in the rebuttal letter that accompanies your revised manuscript. If you need to cite a retracted article, indicate the article’s retracted status in the References list and also include a citation and full reference for the retraction notice.

Response: *Done*

Reviewer #2: The previous comments have been addressed. I have just a few additional comments:

1. The manuscript adheres to PRISMA-ScR reporting guidelines although on lines 79 and 80 of the revised manuscript, it states that ‘the methodology is based on Preferred Reporting Items for a Systematic Review and Meta-analysis of Diagnostic Test Accuracy Studies: The PRISMA-DTA’.

Response: *Thanks for noticing this error. The manuscript and the accompanying reference have been corrected. We realized that our abstract was not compliant with this reporting guideline so minor changes were made to make in the abstract it compliant.*

2. Table 1 is more of a critical appraisal than a quality assessment and the title should reflect that.

Response: *The title was changed as suggested*.

3. The legend states that the purple shape is 'unclear or moderate' while the JBI checklist rates the risk of bias as low, high, unclear or not applicable.

Response: *We omitted ”or moderate” which was an error.*

4. Lines 150 and 151 in Results section, 'hearing loss is first noted in adolescence...' - is this a finding or part of the discussion as the statement is referenced.

Response: *This is a summary of the results in the table. The sentence is now started with “In these reports…”. The reference was removed as it makes it seem like we are talking about a study that is not in the table*.

5. The layout (orientation) of Table 5 needs to change to Landscape as with Tables 4A & B.

Response: *Done*

Reviewer #3: Thank you for this important article highlighting a knowledge deficit in the actual incidence, prognosis, response to therapy of/for congenital syphilis. The recent increase incidence of congenital syphilis in the USA and Canada with unknown incidence in LMICs make filling this knowledge gap even more important as we move forward. I feel the reviewers comments/concerns have been adequately addressed. I do wonder why the authors chose not to have a second reviewer for articles in French. It would be informative to identify which articles were in French but not critical to do so.

Response: *Only one of the authors is fluent in French. In the end, none of the studies in French met the inclusion criteria. Had there been many studies, we would have found another person who was fluent.*

While revising your submission, please upload your figure files to the Preflight Analysis and Conversion Engine (PACE) digital diagnostic tool, <https://pacev2.apexcovantage.com/>. PACE helps ensure that figures meet PLOS requirements. To use PACE, you must first register as a user. Registration is free. Then, login and navigate to the UPLOAD tab, where you will find detailed instructions on how to use the tool. If you encounter any issues or have any questions when using PACE, please email PLOS at [figures@plos.org](mailto:figures@plos.org). Please note that Supporting Information files do not need this step.

Response*: Done*
