# Supplementary material for: Scoping review of hearing loss attributed to congenital syphilis
Source: PLoS One. 2024 Apr 26;19(4):e0302452. doi: 10.1371/journal.pone.0302452 (PMC11051613; doi:10.1371/journal.pone.0302452)
Supplement: Attachment — Submitted filename: Response to reviewers.docx [file pone.0302452.s007.docx]

Response to reviewers:

INTRODUCTION:
Paragraph 2; Lines 3-5: “In 2021 in Canada, the incidence of infectious syphilis in females was 729% higher than in 2017.“
- This statement is incorrect. Authors should check and revise the percentage increase from 7 in 2017 to 96 in 2021.

**Response:** *We decided that it was simpler and clearer to remove percentages and remove maternal Canadian data and present actual data for congenital syphilis for the same years in Canada and the United States. We therefore changed this section to read: “The number of cases of confirmed early CS born to women aged 15-39 years in Canada rose from 17 in 2018 to 117 in 2022 [4]. Trends in the United States (US) mirror this with an increase from 1325 congenital syphilis cases in 2018 to 3755 in 2022 [5].”*

RESULTS
Lines 1&2: Authors please clarify: “Screening led to 154 records for full-text review of which 36 met inclusion criteria (Figure 1).“
- This statement is unclear. Authors need to describe the process of study were selection after removing duplicates to arrive at 154.

**Response:** *Thanks for pointing out this error. It should have been 159 rather than 154 full articles that were pulled. We now added to the text “The figure outlines the reasons for exclusion of other records. “ We can outline the reasons in the text as well if the reviewer thinks that this is necessary.”*

Screening of patients with hearing loss for congenital syphilis
Line 1: “There were 5 studies where patients with hearing loss for were screened for CS.”
- Delete “for” after hearing loss.

**Response:** *Thanks for pointing out this error. It was corrected.*

Table 1: Delete “were” from the subtitles under Type of study column e.g. Children with HL were screened for CS

**Response:** *Done*

Studies with interventions for hearing loss
Line 2: “All were observational and typically included a penicillin with addition of…”
- Authors to revise sentence

**Response:** *This was changed to “All were observational. Most commonly patients were prescribed penicillin with addition of prednisone followed by ACTH if response was poor or transient.”*

DISCUSSION
Opening sentence: “The scoping review shows that the literature on hearing loss due to CS is immature.”
- The word “immature“ in the opening sentence is unclear. Authors should rewrite and clarify.

**Response:** *We changed “immature” to “limited and low quality.”*

Para 1, Last sentence: ”It seems unlikely that a systematic review would answers these questions”
- change “answers” to ‘answer’

**Response:** *Done*

Para 4, Lines 2 & 3: “It is possible that hearing loss started years prior but was not recognized.”
- Authors may also add the following sentence: “Particularly, if the hearing loss is delayed or progressive.”

**Response:** *Thanks for the suggestion. We changed the sentence to read: “It is possible that hearing loss started years prior but was not recognized, particularly, if the hearing loss was slowly progressive.”*

GENERAL
The manuscript requires extensive language edits.

**Response:** *Many edits were made.*
